# Iron chelating properties of Eltrombopag: Investigating its role in thalassemia-induced osteoporosis

**DOI:** 10.1371/journal.pone.0208102

**Published:** 2018-12-03

**Authors:** Francesca Punzo, Chiara Tortora, Maura Argenziano, Maddalena Casale, Silverio Perrotta, Francesca Rossi

**Affiliations:** 1 Department of Woman, Child and General and Special Surgery, University of Campania “Luigi Vanvitelli”, Naples, Italy; 2 Department of Experimental Medicine, University of Campania “Luigi Vanvitelli”, Naples, Italy; University of Louisville School of Medicine, UNITED STATES

## Abstract

Chronic blood transfusions are responsible to cause iron overload, which leads to several complications to end organs and osteoporosis. Iron chelation is needed to remove iron excess and to contain bone-mass loss. Deferasirox is the most recent oral iron chelator that prevents transfusion related iron overload complications. Recently Eltrombopag (ELT) iron chelating properties are emerging. ELT is an agonist at Thrombopoietin receptor, used in treatment of thrombocytopenia. We tested ELT and Deferasirox in iron overloaded osteoclasts from thalassemic patients and donors measuring intracellular iron, TRAP expression and osteoclast activity. We confirmed ELT iron chelation capacity also in bone tissue and a synergic effect when used with Deferasirox. Moreover, having demonstrated its effects on osteoclast activity, we suggest for the first time that ELT could ameliorate bone tissue’s health reducing bone mass loss.

## Introduction

Iron overload is a condition arising from increased iron absorption or as a consequence of chronic blood transfusions. Anemias due to a decrease in RBC production, an increase in cell destruction or chronic blood loss, could require red blood cell transfusion therapy. Beta-Thalassemia Major (TM) and Sickle Cell Disease are examples of chronic conditions that necessitate long-term transfusion therapy to improve life expectancy [1]. This inevitably leads to secondary iron overload that, by free radical production, can cause significant damage to many organs, such as the liver and heart, and to the endocrine system [2, 3]. Iron accumulation in bone tissues is responsible of osteoporosis (OP) [4, 5]. There are no physiological mechanisms to excrete iron excess and to prevent its deposition into end organs, hence it is very important to remove it with a pharmacological approach and limit the serious clinical consequences of its overload [6–10]. Deferasirox (DFX) is the most recent oral iron chelator. It is effective and well-tolerated, once daily, and prevents transfusion related iron overload due to its ability to provide constant chelation coverage and significantly improve patient’s compliance [11, 12]. Recently, we demonstrated that iron overload, through up regulation of Tartrate-resistant acid phosphatase (TRAP) expression, causes over activity of osteoclasts (OCs) in TM and that normal levels of activity can be restored with iron chelation therapy [5]. The improvement in bone mineral density and the reduction of OP reported in a cohort of TM patients treated with DFX [13] have confirmed *in vitro* data.

Eltrombopag (ELT) is a first generation Thrombopoietin receptor (TPO-R) agonist, approved for the treatment of chronic idiopathic thrombocytopenia (ITP) and thrombocytopenia associated with chronic hepatitis C (HCV) in both pediatric and adult patients [14, 15]. In general, HCV infection is associated with a reduced platelet count [16]. Chronic infection with the HCV has been for long time the major co-morbidity in patients with inherited blood disorders such as TM [17]. Another property of ELT is to bind to metal ions and in particular to iron (III). It has been indicated as a powerful iron chelator that decreases ROS-mediated cellular damage and further enhances iron mobilization when combined with clinically available chelators [18]. In pediatric patients with chronic ITP it has been found responsible of inducing iron deficiency anemia [19]. These properties of ELT have been recently demonstrated in human hepatoma cells (HuH7) and rat cardiomyocytes (H9C2) [18].

We tested the iron chelation capacity of ELT for the first time on primary human OCs from thalassemic patients and healthy donors. We investigated the possibility to use ELT also to treat iron overload conditions, either alone or when combined with clinically licensed chelator (DFX), and to test if the use of this drug could also be considered to improve OP condition in chronic transfusion dependent patients.

## Materials and methods

### Patients

Two β-TM patients (mean age 33.1±3.5 years) and two healthy donor women in pre-menopausal age (mean age 32.3±3.9 years), attending the Department of Woman, Child and of General and Specialist Surgery at the Vanvitelli University, were recruited. Patients gave informed written consent on entering the study, which was approved by the University of Campania “Luigi Vanvitelli” Ethics Committee, in accordance with the Declaration of Helsinki. Patients and controls did not present thyroid disease, diabetes mellitus, malabsorption or hypoparathyroidism. Patients received regular transfusions, maintaining pre-transfusion hemoglobin levels at approximately 9.5 g/dL. They did not receive bisphosphonates or steroid/estrogenic treatments for at least 3 years prior to recruitment. Iron overload was assessed by measurement of mean serum ferritin levels over the last 10 years and estimation of liver iron concentration by T2* magnetic resonance imaging. After signing written informed consent, 9 ml of peripheral blood in EDTA were taken from each enrolled individual.

### Human cell cultures

Primary cultures of osteoclasts (OCs) were differentiated from peripheral blood mononuclear cells (PBMCs). PBMCs were isolated by centrifugation over Histopaque 1077 density gradient (Sigma Chemical, St Louis, MO), diluted at 1×10^6^ cells/mL in α-Minimal Essential Medium (α-MEM)) (Lonza, Verviers, Belgium) and supplemented with 10% fetal bovine serum (FBS) (Euroclone, Siziano, Italy), 100 IU/mL penicillin, and 100 g/mL streptomycin (Gibco Limited, Uxbridge, United Kingdom) and L-glutamine. In order to obtain fully differentiated human OCs, the PBMCs were then cultured for 21 days in the presence of 25 ng/mL recombinant human macrophage colony-stimulating factor (rh-MCSF) (Peprotech, London,UK) and 50 ng/mL RANK-L (R&D Systems, Minneapolis, MN). Culture medium was replaced every three days with fresh medium supplemented with the agents described above. Tartrate-Resistant Acid Phosphatase (TRAP) positive cells appeared in culture from day 10.

### RNA isolation, real time PCR

The total RNA was extracted using Quiazol (Quiagen) following the manufacturer’s instructions. EasyScript cDNA Synthesis Kit (abm) was used to synthesize from approximately 500 ng mRNA, the first strand cDNA. The transcript levels of TRAP were detected by RT-qPCR using a CFX96 Real-Time PCR system (Bio-Rad) and using I-Taq Universal SYBR Green Master Mix (Bio-Rad). The cycling conditions were 10 min at 95° C (initial denaturation) followed by 40 cycles of 15 sec at 94° C (denaturation) and 1 min at 68° C (annealing/extension/data collection). The β-Actin gene served as the reference gene for the normalization of the RT-qPCR products.

The linearity and efficiency of the assays were tested over dilutions of input cDNA spanning five orders of magnitude. Assays were performed in technical triplicate. The dissociation curve analysis of amplification products was performed at the end of each PCR reaction to confirm the specificity of the amplification. The 2^-ΔΔCt^ method was used to analyse the data and to obtain the relative gene expression levels compared to the controls.

### Tartrate resistant acid phosphatase (TRAP) assay

TRAP was evaluated as specific OCs biochemical activity marker and quantified using the ACP method (Takara Bio, Japan). After cell fixation (citrate buffer pH 5.4, containing 60% acetone and 10% methanol for 5 min, at room temperature), 50 μL of chromogen substrate solution (naphtol-AS-BI-phosphate substrate/fast red violet LB), mixed with 0.1 volume of sodium tartrate, was added to each well. The TRAP enzyme cleaves the substrate, forming a red azoic dye with purplish red color that can be detected with an optical microscope (Nikon Eclipse TS100, Nikon Instruments, Badhoevedorp, The Netherlands). TRAP(+)-multinucleated-OCs were counted in at least three different wells in each group of treatment. Each experiment included a positive and a negative control to ensure functionality of the assay.

### Iron assay

After treatment with ELT [6μM], DFX [5μM], and ELT [6μM]+DFX [5μM], cell lysates derived from OCs of healthy donor women iron overloaded with FAC [50μM] and thalassemic women, were collected to measure Iron (III). The assay was performed by using the Iron Assay Kit (Abcam, ab83366) according to the manufacturer’s instructions. Briefly, standards and OCs lysates were pipetted into the wells, then incubated with an acidic buffer to allow iron release and then with an iron probe at 25°C for 60 min protected from light. Released iron reacted with the chromogen resulting in a colorimetric (593 nm) product, proportional to the iron amount. The optical density was measured at a wavelength of 593 nm by using the Tecan Infinite M200 (Tecan, Switzerland) spectrophotometer. Iron (II) and Total Iron (II+III) contents of the test samples (nmol/μl) were determined against a standard concentration curve. Iron (III) content of the test samples can be calculated as: Iron (III) = Total Iron (II+III)–Iron (II).

### Protein isolation, western blot

Protein extraction was performed by using RIPA buffer lysis (Millipore, Italia), according to the manufacturer’s instructions. Total protein concentrations were determined with Bradford dye-binding method (Bio-Rad, Hercules, CA, USA). TRAP expression in total lysates from OCs cultures were analysed by Western blot experiments. Twenty micrograms of denatured protein were loaded. Membranes were incubated overnight at 4°C with rabbit polyclonal anti-TRAP (SC-28204, dilution 1:200; Santa Cruz, California, USA) and then with the relative secondary antibody for 1 h. Reactive bands were detected by chemiluminescence (Immobilion western Millipore) on a C-DiGit blot scanner (LI-COR Biosciences). A mouse anti-β-actin antibody (SC-47778, dilution 1:500; Santa Cruz, California, USA) was used to check for identical protein loading and as housekeeping protein. Images were captured, stored and analysed using “Image studio Digits ver. 5.0” software.

### Drugs and treatments

OCs from β-TM patients were differentiated in the presence of ELT [6μM] and DFX [5μM], alone and in combination, from the first day of culture until they were fully differentiated in OCs (day 21). OCs derived from healthy subjects were treated with Ammonium iron (III) citrate (Sigma Aldrich, F5879-100G), FAC [50 μM], from day 7 (second medium change) until they were fully differentiated into OCs. They were then either treated with ELT [6μM] and DFX [5μM] alone and in combination from day 15 until day 21.

### Statistical analysis

Results are expressed as means ± S.D. The experiments were run in triplicate. Statistical analyses were performed using the non-parametric Wilcoxon test to evaluate differences between quantitative variables. A *p* value less than 0.05 was considered statistically significant.

## Results

### Intracellular ferric iron ion concentration is affected by DFX and ELT

**Fig 1:**
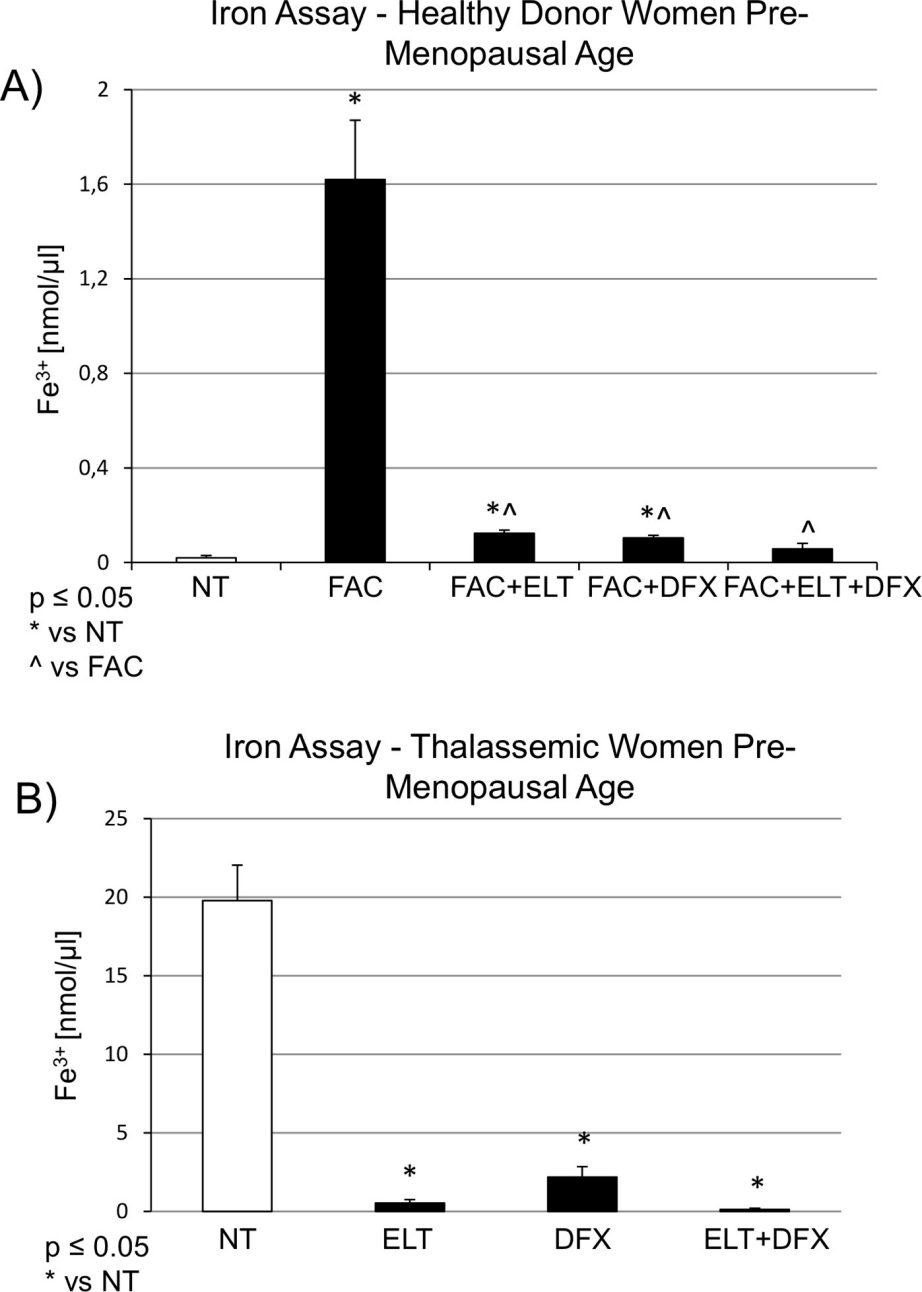
Intracellular ferric iron ion concentration [nmol/μl] measured with the Iron Assay Kit on lysate of osteoclasts from healthy donor and thalassemic women. (A) Osteoclasts (OCs) derived from healthy donor women were treated with Ammonium Iron (III) Citrate, FAC [50μM], from day 7 until they were fully differentiated into OCs (day 21). They were then treated with Deferasirox, DFX [5μM], Eltrombopag, ELT [6μM] from day 15. (B) OCs derived from thalassemic women were treated with Deferasirox, DFX [5μM], Eltrombopag, ELT [6μM] from day 15. The iron concentration (Fe^3+^ nmol/μl) was determined against a standard concentration curve according to the manufacturer’s instructions. The assay was conducted three times. Data are expressed as mean ± SD. The non-parametric Wilcoxon test was used for the statistical analysis. p≤0.05 was considered statistically significant. * indicates p ≤ 0.05 compared to untreated control (NT), ^ indicates p ≤ 0.05 compared to FAC.

To evaluate the iron levels in osteoclasts (OCs) exposed to FAC [50μM], ELT [6μM] and DFX [5μM], alone or in combination, we measured the intracellular ferric iron ion concentration [Fe^3+^]. As expected, FAC [50μM] strongly increased [Fe^3+^] in cell lysates derived from OCs of healthy donor women. ELT [6μM] and DFX [5μM] induced a statistically significant reduction of FAC-induced [Fe^3+^] increase and when used in combination, the reduction was more marked (Fig 1A). A similar trend was observed in cell lysates cultures derived from OCs of thalassemic women, iron overloaded because of their clinical condition. Alone, ELT [6μM] and DFX [5μM] reduced [Fe^3+^] intracellular levels and in combination the reduction observed was even more evident (Fig 1B).

### TRAP expression is affected by DFX and ELT

**Fig 2:**
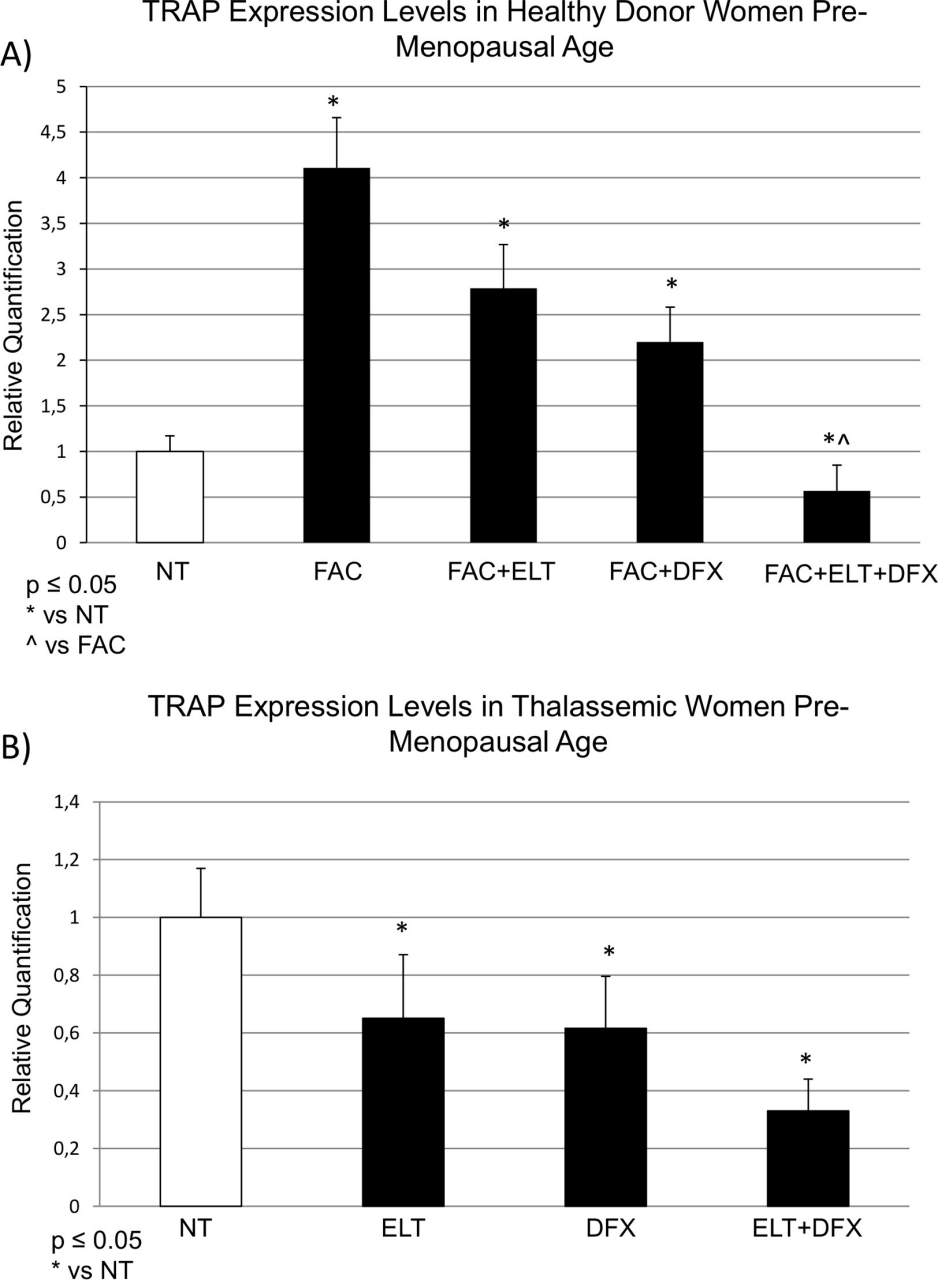
TRAP mRNA expression levels in osteoclasts from healthy donor and thalassemic women. (A) Osteoclasts (OCs) derived from healthy donor women were treated with Ammonium Iron (III) Citrate, FAC [50μM], from day 7 until they were fully differentiated into OCs (day 21). They were then treated with Deferasirox, DFX [5μM], Eltrombopag, ELT [6μM] from day 15. (B) OCs derived from thalassemic women were treated with Deferasirox, DFX [5μM], Eltrombopag, ELT [6μM] from day 15. Results were normalized for the housekeeping gene β-actin and were showed as mean ± SD of three independent experiments on each individual sample. The non-parametric Wilcoxon test has been used to evaluate statistical differences in TRAP expression among groups. * indicates p ≤ 0.05 compared to the untreated control (NT), ^ indicates p ≤ 0.05 compared to FAC.

Molecular levels of TRAP, revealed by RT-qPCR, in OCs cultures derived from healthy donor women, significantly increased after FAC-induced iron overload. The co-application, from day 15, of ELT [6μM] or DFX [5μM] fully abolished FAC-induced TRAP over-expression. Moreover the combined treatment (ELT+DFX) decreased TRAP mRNA levels also compared to untreated OCs of healthy women in pre-menopausal age (Fig 2A).

**Fig 3:**
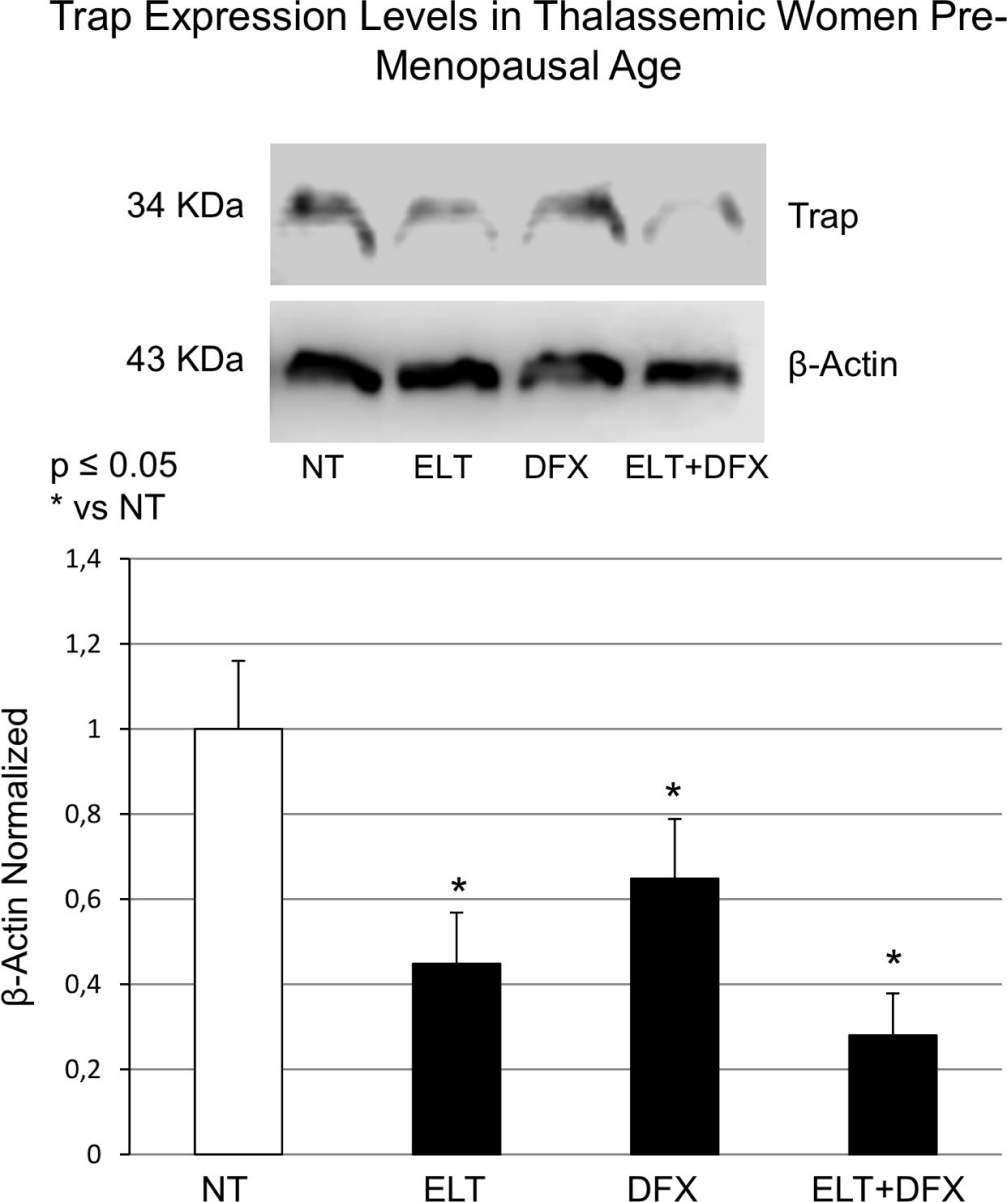
TRAP protein expression levels in osteoclasts from thalassemic women. TRAP protein expression levels in osteoclasts from thalassemic women determined by Western Blot, starting from 20 μg of total lysate, after treatments with DFX [5μM] and ELT [6μM] alone and in combination. Representative photographs were showed. The protein bands were detected using Image Studio Digit Software and the intensity ratios of immunoblots to that of untreated control (NT) taken as 1 (arbitrary unit) were quantified after normalizing with respective loading controls. The histogram shows the relative quantification for TRAP expression normalized for the housekeeping protein β-actin, represented as mean ± SD of individual experiments on each patient. The non-parametric Wilcoxon test has been used to evaluate the statistical differences in protein expression levels. * indicates p ≤ 0.05 compared to NT.

In OCs cultures derived from thalassemic women ELT [6μM] and DFX [5μM], alone, induced a significant reduction of TRAP respect to untreated OCs (naturally iron overloaded because of the patient’s clinical condition) with a striking effect when used in combination (Fig 2B). To confirm molecular data, we measured the TRAP protein expression levels in OCs from thalassemic patients by Western Blot. Also TRAP protein level showed a statistically significant decrease with ELT [6μM] and DFX [5μM] and a much more marked decrease when using the two compounds combined (Fig 3).

### Osteoclasts (OCs) activity and number are affected by DFX and ELT

TRAP was evaluated as specific biochemical activity marker for OCs and quantified using the “TRAP Assay”, which allow us to identify TRAP (+) (stained in purple) multinucleated (n ≥3) OCs. TRAP assay in OCs derived from healthy donor women revealed that *in vitro* iron-overload, using FAC [50μM], induced an increase in both OCs number (about 40%) and size.

**Fig 4:**
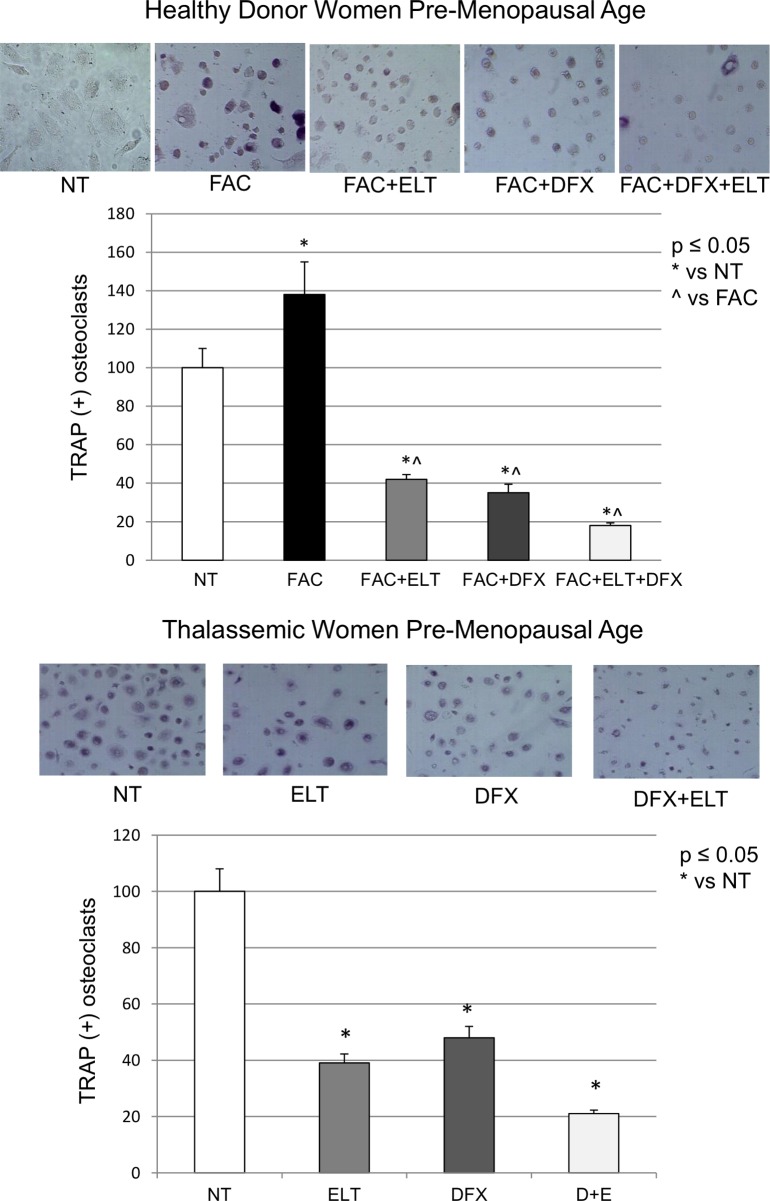
TRAP assay on osteoclasts from healthy donor women and thalassemic women. Osteoclasts (OCs) derived from healthy donor women were treated with Ammonium Iron (III) Citrate (FAC) [50μM], from day 7 until they were fully differentiated into OCs (day 21). They were then treated with Deferasirox (DFX) [5μM] and Eltrombopag (ELT) [6μM] from day 15. OCs derived from thalassemic women were treated with DFX [5μM] and ELT [6μM] from day 15. The representative images of TRAP Assay are displayed. Cells were plated in a 24-well plate. TRAP positive multinucleated OCs are displayed in purple and were counted after 21 days of differentiation, using an a AE2000 inverted microscope at 10x magnification, in at least three different wells for each treatment group. The histogram represents the percentage of TRAP(+) cells respect to the total cell number for each sample. Data have been obtained from three different counts for each group and statistically analysed as mean ± SEM. The non-parametric Wilcoxon test was used for the statistical analysis. p ≤ 0.05 was considered statistically significant. * indicates p ≤ 0.05 compared to untreated control (NT), ^ indicates p ≤ 0.05 compared to FAC.

The co-treatment, from day 15 with ELT [6μM] or DFX [5μM] reversed this effect and the reversion was even more marked when using the two compounds together. Also in OCs cultures derived from thalassemic women, TRAP positive OCs were significantly reduced after treatment with ELT [6μM] and DFX [5μM] and the reduction was doubled when using ELT [6μM] and DFX [5μM] together (Fig 4).

## Discussion

Osteopenia and osteoporosis (OP) are responsible for morbidity in 40–50% of adult patients with β-thalassemia major (TM), leading to chronic back pain, bone fractures, skeletal deformities and nerve compression, reducing patients’ life quality [20]. The molecular and cellular mechanisms responsible for the pathogenesis of bone resorption in TM remain poorly understood. We demonstrated that iron overload, through up-regulation of TRAP expression, causes overactivity of osteoclasts (OCs) in TM and that normal functional levels can be restored with chelation therapy, suggesting the possibility that oral chelation could alleviate TM-associated OP [5]. TM patients are exposed to an high risk of hepatitis C (HCV) due to the frequent blood transfusions [17]. HCV infection causes an abnormal thrombocyte function and has been shown to be associated with a low platelet count [16].

Eltrombopag (ELT) is an orally bioavailable small molecule, Thrombopoietin Receptor (TPO-R) agonist, approved for the treatment of idiopathic thrombocytopenia (ITP) and thrombocytopenia associated with chronic HCV in both pediatric and adult patients [14, 15].

ELT is also structurally similar to molecules that have been developed for oral iron chelation therapy. Recently Vlachodimitropoulou *et al*. have demonstrated that ELT has an high binding constant for iron(III) and it is a powerful iron chelator that mobilizes cellular iron from human hepatoma cells (HuH7) and rat cardiomyocytes (H9C2), rapidly decreasing intracellular ROS and its iron chelation capacity is enhanced when combined with clinically available chelators. They demonstrated that ELT mobilizes iron citrate species faster than oral cheletors, but rapidly donates the chelated iron to Deferasirox (DFX), probably with a shuttling mechanism [18].

In this study we tested the iron chelation properties of ELT in primary cultures of OCs from healthy donors and TM patients and its potential to reduce OCs activity, usually increased in patients with iron overload, as consequence of chronic blood transfusions.

Therefore we evaluated the effects of ELT, alone and in combination with the most recent and best tolerated iron chelating compound, DFX, on intracellular ferric iron concentration and on OCs number, size and activity. As previously demonstrated [5], in OCs from TM patients, TRAP expression and activity are increased in presence of iron overload, confirming its role in the dysregulation of bone metabolism. Accordingly, we found that FAC iron overloaded OCs, derived from healthy subjects, presented an increase of the intracellular ferric iron ion (Fe^3+^) concentration, TRAP expression and OCs number, size and activity. These effects were completely abolished by the chelating agents ELT and DFX. And, it is very important to underline, the effect observed was drastically increased by the combination of ELT with DFX, suggesting a greater intracellular iron mobilization due to a synergism in the action of the two molecules combined. It is not known if ELT might increase thrombotic predisposition in TM or other chronic blood transfusion dependent patients, but it is conceivable to hypothesize a possible use of ELT combined with DFX in HCV positive TM patients.

Although this study has been conducted only on few samples and in vivo experiments will be necessary to assess whether these *in vitro* effects can be translated to the clinical practice, this study is the first one conducted on primary human cells and provides a first and timely evidence of efficacy of ELT as iron chelator in bone tissue. Moreover we hypothesize a possible therapeutic application of this compound to restore bone health in TM and in other chronic blood transfusion dependent patients.

## Supporting information

S1 Fig Uncropped WB image β-Actin. β-Actin protein expression in osteoclasts from thalassemic women determined by Western Blot, starting from 20 μg of total lysate, after treatments with DFX [5μM] and ELT [6μM] alone and in combination. The protein bands were detected using Image Studio Digit Software and the intensity has been used as loading control to normalize TRAP protein expression.
(TIF)

S2 Fig Uncropped WB image TRAP. TRAP protein expression in osteoclasts from thalassemic women determined by Western Blot, starting from 20 μg of total lysate, after treatments with DFX [5μM] and ELT [6μM] alone and in combination. The protein bands were detected using Image Studio Digit Software and were quantified after normalizing with respective loading controls.
(TIF)
