# Supplementary figures and images for: Iron chelating properties of Eltrombopag: Investigating its role in thalassemia-induced osteoporosis
Source: PLoS One. 2018 Dec 3;13(12):e0208102. doi: 10.1371/journal.pone.0208102 (PMC6277068; doi:10.1371/journal.pone.0208102)

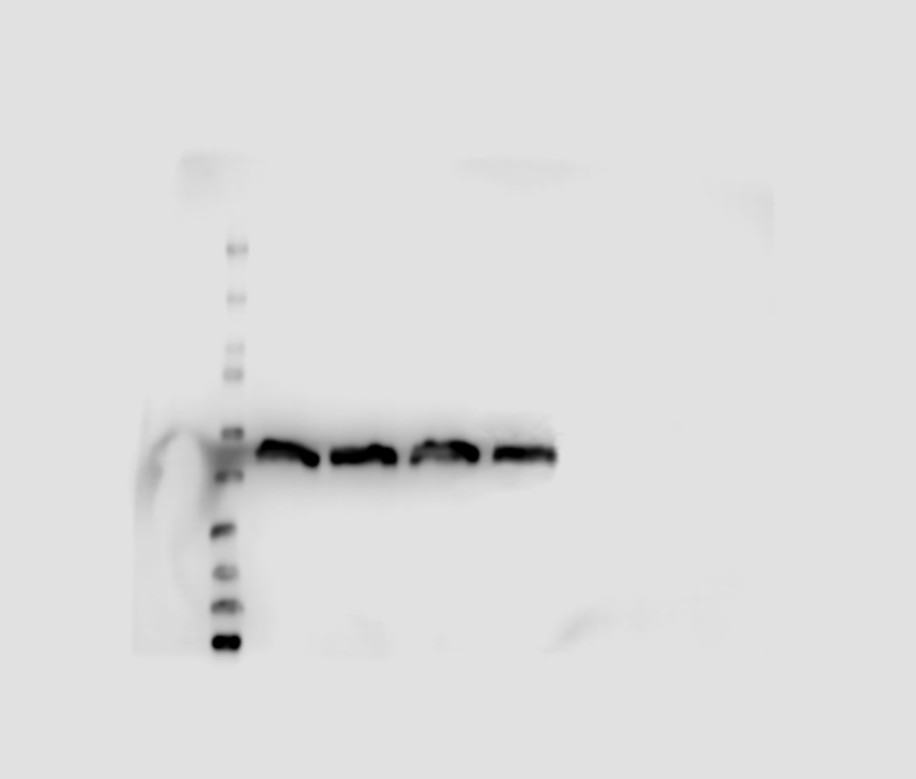

Supplement: S1 Fig — β-Actin protein expression in osteoclasts from thalassemic women determined by Western Blot, starting from 20 μg of total lysate, after treatments with DFX [5μM] and ELT [6μM] alone and in combination. The protein bands were detected using Image Studio Digit Software and the intensity has been used as loading control to normalize TRAP protein expression. (TIF) [file pone.0208102.s001.tif]

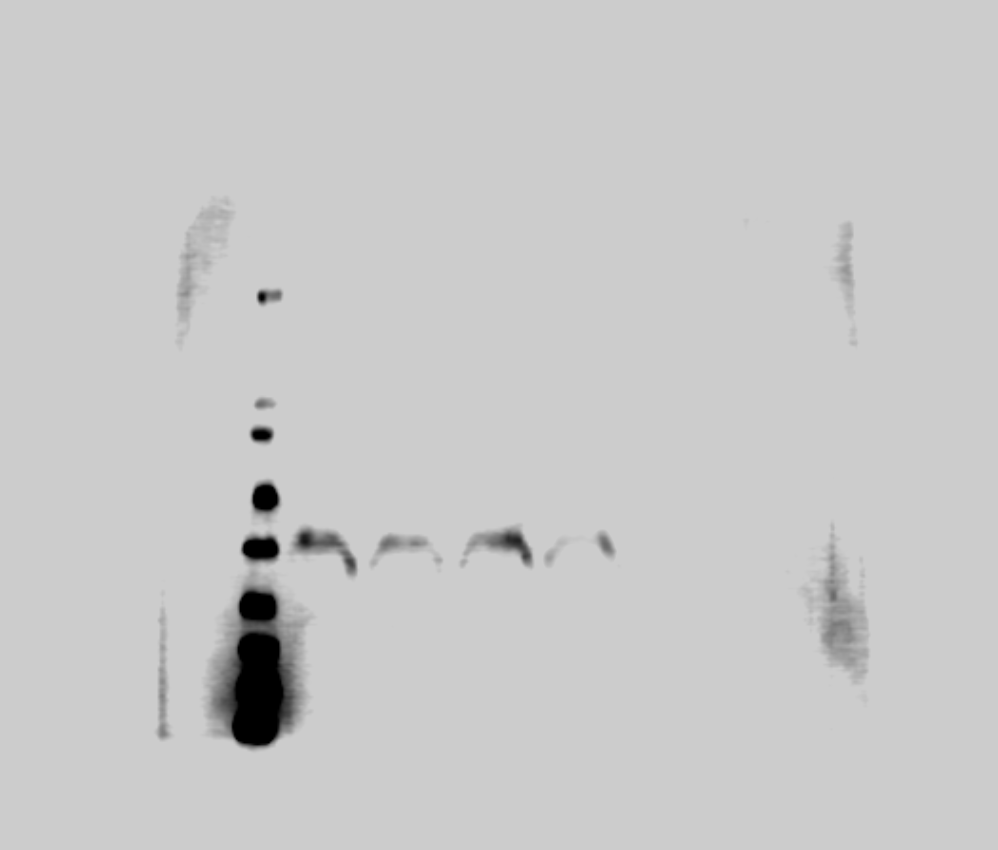

Supplement: S2 Fig — TRAP protein expression in osteoclasts from thalassemic women determined by Western Blot, starting from 20 μg of total lysate, after treatments with DFX [5μM] and ELT [6μM] alone and in combination. The protein bands were detected using Image Studio Digit Software and were quantified after normalizing with respective loading controls. (TIF) [file pone.0208102.s002.tif]
